# Patients with suspected allergic reactions to COVID‐19 vaccines can be safely revaccinated after diagnostic work‐up

**DOI:** 10.1002/clt2.12044

**Published:** 2021-07-27

**Authors:** Trine Holm Rasmussen, Charlotte Gotthard Mortz, Torbjorn Kabel Georgsen, Helene Marlies Rasmussen, Henrik Fomsgaard Kjaer, Carsten Bindslev‐Jensen

**Affiliations:** ^1^ Department of Dermatology and Allergy Center Odense Research Centre for Anaphylaxis (ORCA) Odense University Hospital Odense Denmark

**Keywords:** allergy, COVID‐19 vaccine, diagnostic test, excipients, revaccination

## Abstract

**Background:**

When initiating the Danish vaccination program against COVID‐19, the incidence of anaphylaxis was estimated to be 10 times higher compared to other virus‐based vaccines. In this study, we present data on patients referred with suspected allergic reactions to COVID‐19 vaccines. The main purpose of the study is to investigate the incidence and severity of the allergic reactions, and to evaluate the safety of revaccination.

**Methods:**

All patients in the region of Southern Denmark with case histories of allergic reactions to COVID‐19 vaccines in a defined period are included in this study. Diagnostic work up consisted of a detailed case history, evaluation of Brighton level of diagnostic certainty and World Allergy Organization grade of anaphylaxis and skin prick testing‐ and basophil histamine release testing with COVID‐19 vaccines and relevant drug excipients. Patients were revaccinated at the Allergy Center when possible.

**Results:**

Sixty‐one patients are included in this study. In 199,377 doses administered, nine patients fulfilled the criteria of anaphylaxis when using the Brighton Criteria (incidence being 45 per million). Of 55 patients with reactions to the first dose, 52 patients were revaccinated without adverse reactions. We found no proven cases of immediate anaphylaxis due to COVID‐19 vaccines. By skin prick test, we diagnosed three patients with drug excipient allergy and further a patient with mastocytosis was found.

**Conclusions:**

Anaphylactic reactions to COVID‐19 vaccines are rare and the incidence is similar to what is seen with other virus‐based vaccines. Revaccination is safe in the majority of patients; however, allergological evaluation is important since some prove to have drug excipient allergy.

## 1 INTRODUCTION

Virus‐based vaccines are expected to elicit anaphylactic reactions with a frequency of 1.3:1,000,000.^1^ Based on data from the United States,^2^ the COVID‐19 vaccines are suspected to elicit reactions more frequently, estimated by the Danish authorities to 1:100,000.^3^ For safety reasons, we therefore set up a diagnostic routine prior to vaccination in order to investigate reactions to COVID‐19 vaccines and to diminish reactions to COVID‐19 vaccines by identifying patients with high risk of reacting to vaccines. We thus decided to classify citizens into four groups, the first being citizens experiencing an allergic reaction to the COVID‐19 vaccine. These patients are described here. The second group consists of all Danish patients with a diagnosis of systemic mastocytosis, where an even higher frequency of anaphylaxis may be expected, although solid data are missing.^4^ All Danish patients with mastocytosis will be vaccinated in our department, where full anaphylaxis staff and equipment are available. The third group consists of approximately 25 patients with an already established diagnosis of allergy to drug excipients, mostly macrogols/polyethylene glycols (PEG), or polysorbates. Finally, patients with a previous reaction to a virus‐based vaccine, or to parenteral drugs, containing PEG or polysorbate, are referred to the Allergy Center for evaluation prior to COVID‐19 vaccination. Data from the three latter groups will be presented elsewhere.

## 2 METHODS

The vaccination program for citizens in the region of Southern Denmark is organized in seven centers, supplemented with mobile vaccination clinics for nonambulant people. Health professionals are vaccinated in the hospitals. Our department is responsible for vaccinating the staff of Odense University Hospital (8000 persons) and the citizens from the major part of the island Funen (500,000 citizens). All serious immediate reactions to a COVID‐19 vaccine are treated initially in the vaccination center and afterwards in the nearest acute ward. Delayed reactions are treated either in the acute ward, other medical departments, or by the patient's general practitioner (GP).

All adverse reactions to COVID‐19 vaccines are reported to the Danish Medicines Agency, but due to restrictions based on GDPR legislation, the authorities are not permitted to refer patients directly for diagnostic evaluation. Instead, patients from our entire region (approx. 1.2 million inhabitants) with a suspected allergic reaction to a COVID‐19 vaccine were referred to the Allergy Center at Odense University Hospital from the acute wards, other hospital departments, and from the GPs.

In this study, we included all patients referred with a case history of allergic reaction to a COVID‐19 vaccine, paying their first visit to the Allergy Center in the period from January 11, 2021 to April 14, 2021. The patients, seen in this period, had a suspected allergic reaction to a COVID‐19 vaccine in the period from December 27, 2020 to March 20, 2021. Up to this date approximately 200,000 doses of COVID‐19 vaccines were administered to the population of Southern Denmark (156,000 doses of the Pfizer‐BioNTech vaccine (PB‐vaccine), 14,000 doses of the Moderna vaccine (M‐vaccine), and 30,000 doses of the AstraZeneca Vaccine (AZ‐vaccine)).^5^

For all patients in the Allergy Center diagnostic work up consisted of a detailed case history, a detailed past history including concomitant allergies, vaccination history, and present medications obtained from the Danish National Prescription Registry (www.sundhed.dk). The events during the adverse reaction to COVID‐19 vaccination were meticulously recorded including timing, sequence of symptoms and signs, presence of cofactors such as exercise, drugs or infections, together with evaluation of Brighton level^6^, ^7^ and WAO grade^8^ used in classification of anaphylaxis, Table 1. Skin prick testing (SPT) in duplicate with the available vaccines and drug excipients (PB‐vaccine, M‐vaccine, AZ‐vaccine [using residual remnants in the original vials, obtained daily from our in house vaccination center for hospital staff], DMG‐PEG 2000 [content in the M‐vaccine, Merck, concentration 20%], ALC‐0159 PEG 2000 [content in the PB‐vaccine, Sinopeg, China, concentration 20%], all, diluted in sterile water, and Polysorbate 80 [content in the AZ‐vaccine] and 20 [Merck, concentration 100%], together with PEG 2000 [Thermo Fisher, concentration 50%], PEG 3000, 6000 [Merck, concentration 50%], PEG 3350 [Movicol junior Neutral®, Norgine B.V., concentration 100%], PEG 20.000 [Merck, concentration 10%]) were performed with a 1 mm ALK lancet at the volar surface of the forearm. Histamine solution (10 mg/ml) and saline were used as positive and negative control, respectively.^9^, ^10^ The size of the resulting wheals was recorded after 15 min and wheal size was measured on the longest and shortest perpendicular axis, the numbers were added and divided by two (mean wheal diameter). Wheals ≥3 mm larger than the negative control were considered positive. Blood was drawn for measurement of specific IgE to latex protein and chlorhexidine (Thermo Fisher Scientific), and for basophil histamine release (BaHR) (www.Reflab.dk), using the same allergens as in SPT in six dilutions.^11^ BaHR was only considered significantly positive, when a bell shaped curve with at least two positive values above baseline was obtained. BaHR with release above 15 ng/ml, not fulfilling these criteria, were considered marginally positive, but treated as negative when evaluating possibility for revaccination. S‐tryptase level (Thermo Fisher Inc) and c‐KIT mutation^12^ were measured. c‐KIT mutation in blood was detected by using real‐time qPCR assay.^12^ Baseline level of s‐tryptase >12 μg/L was considered elevated, and a s‐tryptase level, measured following the acute allergic reaction, was considered elevated from baseline, when exceeding baseline s‐tryptase x 1.2 + 2^8^.

**TABLE 1:** The Brighton and the WAO Criteria of anaphylaxis

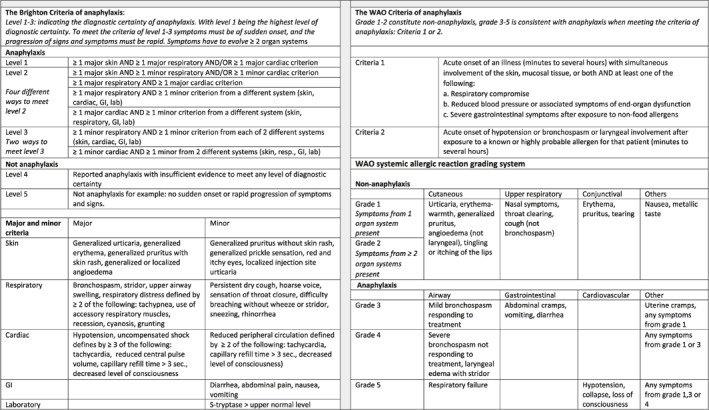

*Note:* The Brighton Criteria of anaphylaxis^6^, ^7^ including the levels of diagnostic certainty are described here together with the WAO Criteria of anaphylaxis and the WAO systemic reaction grading system.

Based on case history and the outcome of the diagnostic tests, patients were allocated to receive the second vaccination at the Allergy Center with the most suitable vaccine or to await the launching of alternative COVID‐19 vaccines with a suitable profile (vaccine and excipients).

Comparison between Brighton level and WAO grade was performed using the nonparametric Spearman correlation test. Statistical analysis was performed with STATA/SE 16.0 (Stata Corporation).

### 2.1 Ethics

In this study, we report the results of our standard diagnostic work up for patients with suspected allergic reactions to a COVID‐19 Vaccine. Written informed consent was obtained from all patients. The study was approved by the Danish Data Protection Agency (Journal nr.: 20/62102) and the Ethics Committees (Report nr.: Covid‐21/209, nr. 50).

## 3 RESULTS

Sixty‐one patients were referred to the Allergy Center after a case history of an allergic reaction to a COVID‐19 vaccine: 30, 6, and 25 patients who were vaccinated with the PB‐, M‐ and AZ‐vaccine, respectively. This cohort includes 54 females and 7 males: age ranging from 18 to 88 years (median 46 years).

In Table 2, patients are arranged according to Brighton level,^6^ time of onset of the adverse reaction, the patient's primary treatment place, and the treatment administered. Nine patients were meeting the criteria of anaphylaxis according to the Brighton criteria (level 1 through 3).

**TABLE 2:** Brighton level of severity and timing of reactions for 61 patients with suspected allergic reactions to COVID‐19 vaccines

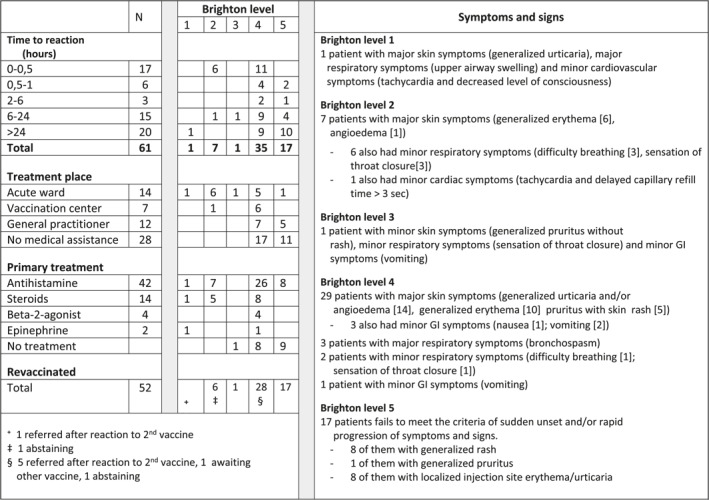

*Note:* Acute treatment and breakdown of symptomatology in the five Brighton levels are also presented together with an overall response to revaccination. Brighton levels 1 through 3 have high but reducing level of certainty for anaphylaxis, whereas level 4 and 5 do not meet the anaphylaxis criteria, although three patients demonstrate major respiratory symptoms (bronchospasm).

In Table 3, the patients are arranged according to onset and severity of reactions using the WAO criteria of anaphylaxis.^8^ Twenty‐six patients had an adverse reaction within 6 h, Tables 1 and 2. Except from two cases of asthma attacks, in patients with known asthma, almost all patients were suffering from skin symptoms. The major skin symptom was flushing and often coupled with subjective symptoms, mostly discomfort, sensation of throat tightness, shortness of breath, and nausea. Going through the patients' histories and available photos, flushing was often misclassified as urticaria at the primary treatment location. Thus, only one patient, with onset of the adverse reaction within 30 min after first vaccine (AZ), had generalized urticaria.

**TABLE 3:** WAO grade of anaphylaxis and timing for 61 patients with suspected allergic reactions to COVID‐19 vaccines

Time to onset (Hours)	0‐0.5					0.5‐1					2‐6					6‐24					>24						Σ
Mean age years (range)	43 (29‐59)					54 (26‐86)					35 (20‐45)					43 (19‐74)					52 (18‐88)						47 (18‐88)
**WAO grade**	**0**	**1**	**2**	**3**	**Σ**	**0**	**1**	**2**	**3**	**Σ**	**0**	**1**	**2**	**3**	**Σ**	**0**	**1**	**2**	**3**	**Σ**	**0**	**1**	**2**	**3**	**5**	**Σ**	
**N**	0	8	6	3	17	1	4	0	1	6	0	2	0	1	3	1	10	0	4	15	6	11	1	1	1	20	61
**Symptoms and signs in patients**																											
*Skin*																											
Urticaria		1			1							1			1		4			4		3			1	4	10
Rash							1			1		1			1		2		1	3		6				6	11
Itch		3	1	1	5		1			1		1			1		5		1	6		3	1			4	17
Flushing		7	4	2	13		1			1							1		1	2					1	1	17
Angioedema		1	2		3		1			1							3		1	4		3			1	4	12
Tingling lips		2	2	1	5																						5
Localized skin reaction		2			2	1	1		1	3						1			1	2	6		1			7	14
*Respiratory system*																											
Bronchospasm				1	1				1	1														1		1	3
Shortness of breath			1	1	2									1	1				1	1							4
Stridor																											
Upper respiratory symptoms			3		3														1	1			1		1	2	3
*Gastrointestinal system*																											
Vomiting																			3	3		1				1	4
Diarrhea																			1	1							1
Nausea			2	1	3																						3
*Cardiovascular system*																											
Hypotension																											
Collapse																									1	1	1
**Past medical history**																											
Asthma			1		1		1		1	2				1	1	1	2			3	1	2	1	1		5	12
Hay fever		3	2		5	1	3		1	5		1		1	2	1	3			4	3	3				6	22
Urticaria/angioedema		1			1		1			1							4		1	5	1	4				5	12
Eczema		2	2		4		2			2		1			1		2			2	1	2	1	1		5	14
Food allergy																											
Drug allergy		1			1												1			1			1			1	3
**Cofactors**																	1			1					1	1	2
**Vaccination status**																											
Vaccinations past 5 years (mean)		2.0					2.3					2.7					1.3					1.2					1.6
Vaccinations range		0‐5					1‐5					0‐4					0‐7					0‐6					0‐7

*Note:* Concomitant atopy, nonatopic drug allergy and chronic spontaneous urticaria/angioedema, presence of cofactors during the reaction, and history of previous vaccinations are also presented. WAO grade 1–2 constitute nonanaphylaxis, grades 3–5 is consistent with anaphylaxis when meeting the criteria of anaphylaxis: Criteria 1 or 2 as given in Table 1.

In this cohort only 1 patient (N60) fulfilled the anaphylaxis Brighton level 1 criteria. The incident took place 40 (!) hours after the second vaccination with the PB‐vaccine. The patient woke up at night suffering from headache. He consumed 600 mg of Ibuprofen® (NSAID) and immediately developed upper airway swelling, urticaria, and fell unconscious. He received prehospital treatment with i.v. clemastine (Tavegyl ® 2 mg) and methylprednisolone (Solu‐Medrol® 125 mg) and was admitted to the acute ward where he further received adrenaline inhalation and was hospitalized until the next day. S‐tryptase level was significantly increased, 8.25 μg/L (basal level 3.01 μg/L). Oral challenge test to ibuprofen® (cumulated 810 mg), 37 days later was negative. He is currently undergoing further diagnostic work up.

Eight patients met the Brighton criteria level 2 or 3 after the first COVID‐19 vaccination, Table 1; six of them with an adverse reaction within 30 min. Two patients had late onset reactions. Seven patients were primarily treated at the acute ward. No increase in s‐tryptase level during reaction was found.

Thirty‐five patients had late onset adverse reactions 6 h or more after vaccination, including patient (N60) with severe anaphylaxis (Tables 2 and 3). Most patients suffered from skin symptoms: Urticaria, rash, itch, angioedema, and localized injection site reactions, for some coupled with subjective respiratory symptoms or gastrointestinal symptoms. In addition, a case of asthma attack in an asthma patient was seen. In the vast majority of cases, urticaria, angioedema, and rash were photo documented by the patients.

No patients reported a prior history of allergic reactions to vaccines, and the cohort had in average received 1.6 virus‐based vaccines during the last 5 years, Table 3. Thirty‐nine patients (64%) were or had been suffering from at least one atopic disease: Hay fever 36%, eczema 23%, and/or asthma 20%, and 20% had chronic spontaneous urticaria/angioedema. We did not find a correlation between atopy in the patient and severity nor timing of the reaction to COVID‐19 vaccine, Table 3.

### 3.1 Diagnostics and revaccination

**FIGURE 1:**
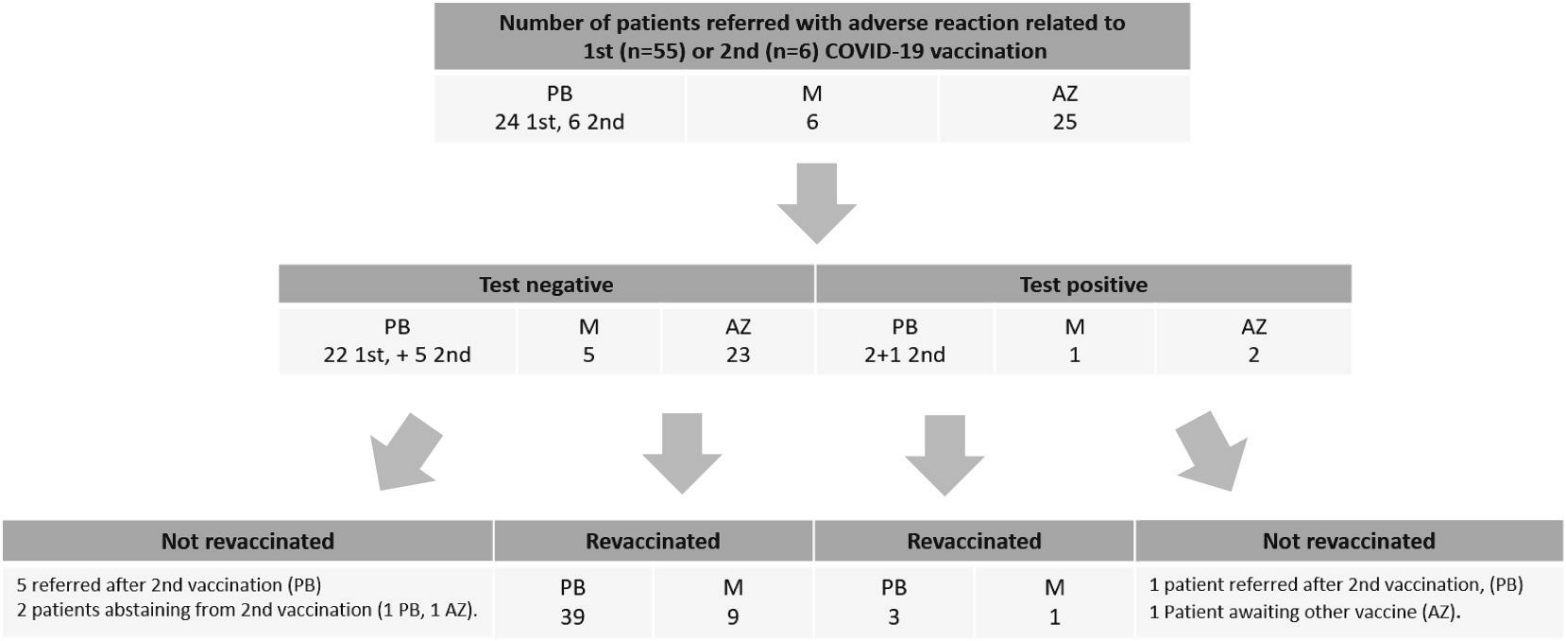
Schematic presentation of the outcome of revaccination. PB, Pfizer‐BioNTech; M, Moderna; AZ, AstraZeneca. Note that six patients were referred after reaction to the second vaccination

Figure 1 and Table 4 present an overview of the diagnostic work up, where in SPT and BaHR testing, 55 patients were negative (50 patients tested after first dose of vaccine), and six patients proved test positive (five patients were tested after first dose of vaccine), Table 4.

**TABLE 4:** Patients with positive test results or cofactors involved in adverse reactions to COVID‐19 vaccination

				Brighton	1: Previous exposure to PEG‐containing laxatives/depo steroid injections		SPT	BaHR		
ID	Vaccine	Sex	Age	level	2: Verified drug allergy	Cofactor	(Positive)	(Positive)	Other tests	Outcome after testing
N1	PB1	M	54	4	1: No exposure	ACE inhibitor				Tolerated revaccination
					2: None					
N60	PB2	M	59	1	1: No exposure	NSAID			Oral challenge test with NSAID (Ibuprofen ^®^): Negative	Referred after reaction to 2^nd^ vaccination
					2: None	(Ibuprofen ^®^)				
N30	AZ1	F	19	4	1: No exposure		PEG 2000, PEG 3000	Pfizer‐BioNTech	Oral challenge test with PEG 3350	Awaiting alternative vaccine
					2: Zaditen^®^ eyedrops: Contact dermatitis		DMG‐PEG 2000,	Moderna	(Movicol ^®^) Positive	
							Polysorbate 20 and 80,			
							Moderna, AstraZeneca			
N23	M1	F	88	5	1: Tolerates Movicol^®^		DMG‐PEG 2000			Tolerated revaccination.
					2: None					
N61	PB2	F	59	4	1: Tolerates Depo‐Medrol ^®^		DMG‐PEG 2000			Referred after reaction to 2^nd^ vaccination
					2: Movicol^®^: case history of flushing, nausea and dizziness		ALC‐0159‐ PEG 2000			
N2	PB1	F	22	2	1: Tolerates Movicol ^®^ and Depo‐Medrol ^®^			Pfizer‐BioNTech		Tolerated revaccination
					2: None					
N12	PB1	F	43	5	1: Tolerates depo steroid injections: Unknown drug.			Moderna		Tolerated revaccination
					2: None					
N53	AZ1	F	48	2	1: No exposure			AstraZeneca		Tolerated revaccination
					2: None					
N52	PB1/PB2	F	39	4	1: No exposure 2: None				KIT D816V mutation‐positive cells (0.05 %).	Referred after reaction to 1^st^ and 2^nd^ vaccination.
									S‐tryptase level: 1.82 µg/L	
N40	PB1	M	70	5	1: No exposure				Baseline level s‐tryptase: 24.6 µg/L	Tolerated revaccination.
					2: None				No detectable KIT D816V mutation positive cells	
N57	M1	F	55	4	1: No exposure				Baseline level s‐tryptase: 22.3 µg/L	Tolerated revaccination.
					2: Penicillin and NSAID				No detectable KIT D816V mutation positive cells	

*Note:* Positive outcome of testing in single patients. Previous reactions to drugs containing high levels of PEGs including depot steroid solutions for injection and electrolyte lavage solutions (e.g., Depo‐Medrol^®^ and Movicol^®^), and positive results in SPT‐, and BaHR testing. Other positive findings during work up are also presented together with the outcome of revaccination.

Abbreviations: BaHR, basophil histamine release; PEG, polyethylene glycols; SPT, skin prick testing.

Of the 55 patients with an adverse reaction to the first COVID‐19 vaccine we have successfully revaccinated 52 patients (revaccinated the patient without occurrence of any symptoms or signs), including seven of the eight patients meeting Brighton level 2 or 3 (the ninth patient [Brighton level 1] was referred after second vaccination). Six patients were referred after an adverse reaction to the second COVID‐19 vaccination (three had reactions to both vaccinations), Figure 1. Due to the exclusion of the AstraZeneca vaccine from the Danish vaccination program, 22 test negative patients, reacting to the COVID‐19 AZ‐vaccine were successfully revaccinated with PB‐vaccine (*n* = 18) or M‐vaccine (*n* = 4). Figure 1. The patient, developing generalized urticaria within 10 min after first vaccination with AZ‐Vaccine, was test negative and was successfully revaccinated with the PB‐vaccine.

In addition to patient (N60), cofactors, (ACE inhibitor), may have played a role in patient (N1) developing angioedema 4 days after first COVID‐19 vaccination, Table 4. He was successfully revaccinated after discontinuation of the ACE inhibitor.

In one patient (N52), we found a mutation in the KIT D816 V gene in peripheral blood.^12^ The patient was referred after second PB‐vaccine, having experienced urticaria after both vaccinations. She had no previous history of anaphylaxis or urticaria/angioedema. SPT and BaHR testing were negative. She is now undergoing confirmatory diagnostic work up for mastocytosis.

In two patients (N40 and N57), we found raised level of s‐tryptase without a mutation in the KIT D816 V gene in peripheral blood. Patient (N40) had had a kidney transplant. In 2016, we diagnosed patient (N57) with drug allergy towards penicillin and NSAID (positive challenge tests). S‐tryptase level was already elevated in 2016, where bone marrow biopsy was performed without pathological findings. SPT and BaHR testings were negative in both patients, who were successfully revaccinated.

One patient (N30) was test positive in both SPT and BaHR testing. She suffered from generalized urticaria 16 h after vaccination with the AZ‐vaccine and had a history of contact dermatitis to ketotifen (Zaditen®) eyedrops. In the three previous years, she had experienced episodes of generalized urticaria. SPT and BaHR testing showed convincing positive results to all drug excipients and vaccines. Oral challenge test with PEG 3350 (Movicol ®) was positive (generalized urticaria) after ingestion of 6.6 g. She is now awaiting launching of a vaccine containing other excipients.

Two patients (N23 and N61) were positive in the SPT testing only. One patient (N 23) was hospitalized 3 days after first COVID‐19 vaccination with the M‐vaccine, due to severe skin symptoms commencing 36 h after vaccination. She was successfully revaccinated at the Allergy Center after negative diagnostic work up. Within few minutes after second PB‐vaccine in the primary vaccination center, the second patient (N61) developed flushing/generalized erythema, discomfort, nausea, dizziness and hypertension without urticaria or angioedema. She was admitted to the acute ward and treated with clemastine (Tavegyl ® 2 mg), and hospitalized until the next day. S‐tryptase level during attack was not elevated. In 2016, she had a similar reaction to PEG 3350 (Movicol®). SPT tests were positive to DMG‐PEG‐2000 and ALC‐0159 PEG 2000 after 2 h. She is now undergoing further investigations for allergy to polyethylene glycols.

Three patients were marginally positive in the BaHR testings (N2, N12, and N53). Patient (N2) had an adverse reaction to the PB‐vaccine: 16 h after first vaccination, she developed angioedema affecting her face, and shortness of breath. She was admitted to the acute ward and treated with i.v. methylprednisolone (Solu‐Medrol ® 80 mg) and clemastine (Tavegyl ® 2 mg). Fifty days prior to vaccination, she was infected with COVID‐19. Nine days after COVID‐19 vaccination, the BaHR testing to the PB‐vaccine was marginally positive. Four months later, repeated diagnostic work up was negative. Patient (N53) had an adverse reaction within 10 min after vaccination with the AZ‐vaccine, experiencing mild angioedema affecting the face, generalized itching, and sensation of throat closure. She was admitted to the acute ward and treated with Clemastine (Tavegyl® 2 mg). The BaHR testing was marginally positive to the AZ‐vaccine. One patient (N12) had an adverse reaction to the PB‐vaccine 16 h after vaccination, having localized urticaria at the trouser lining. The BaHR testing was marginally positive to the M‐vaccine. All three were successfully revaccinated, patient N53 with the PB‐vaccine.

**FIGURE 2:**
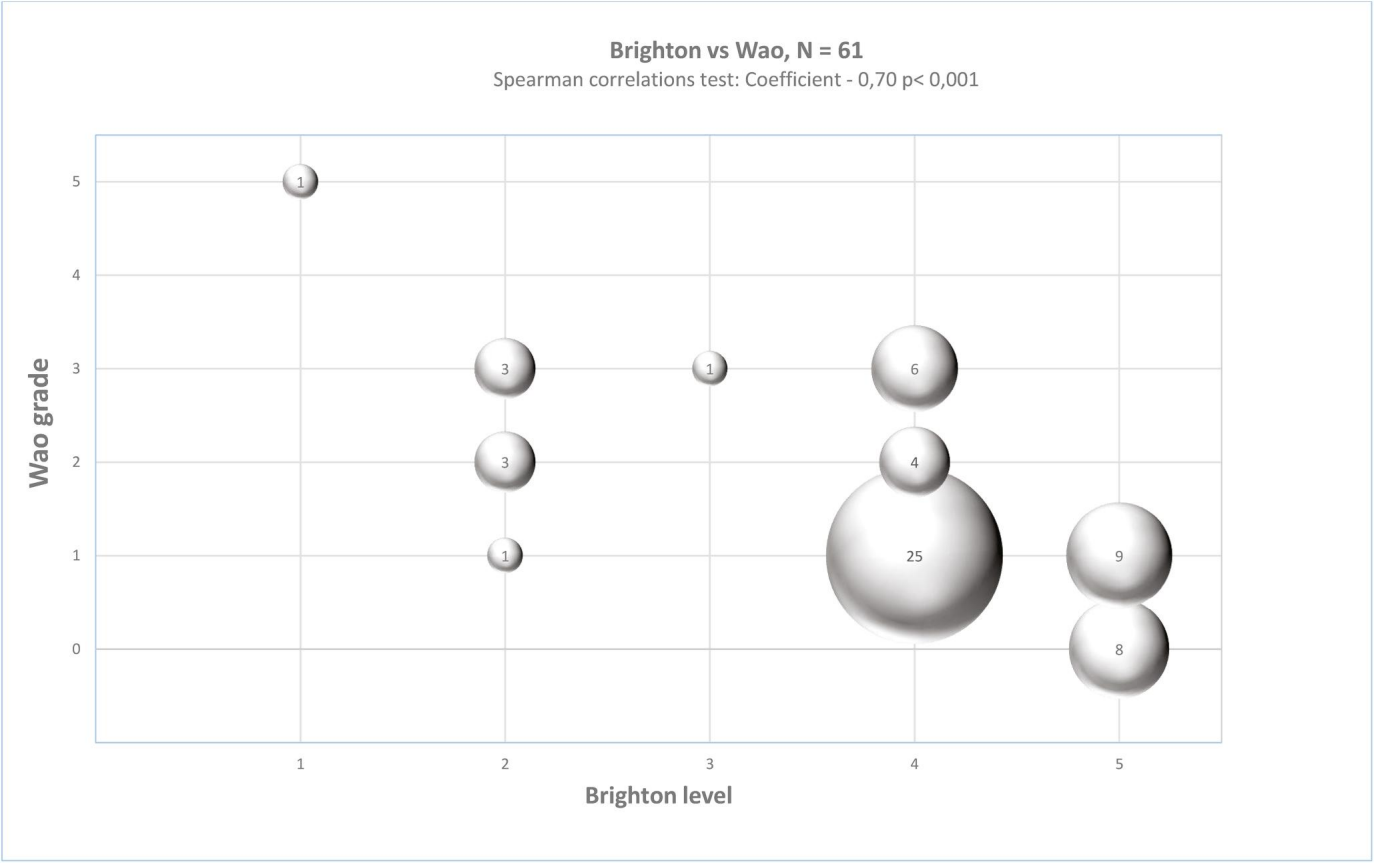
Correlation between Brighton and WAO scoring systems for anaphylaxis. A significant correlation is presented. Spearman correlation coefficient −0.70; *p* < 0.01

The authorities classify patients with reported allergic reactions according to the Brighton criteria.^6^ Allergologists are commonly applying the World Allergy Organization (WAO) anaphylaxis criteria.^8^ In Figure 2, we present the significant correlation (Spearman coefficient −0.70; *p* < 0.01), between the Brighton level and the corresponding WAO grade, for our 61 patients. Four of seven patients with a Brighton level of 2, however, are not classified as anaphylaxis according to the WAO criteria, whereas six out of 35 patients, with a Brighton level of 4, are classified as anaphylaxis according to the WAO criteria. According to the Brighton criteria,^6^ nine patients were meeting the criteria for anaphylaxis level 1 through 3, and eleven patients were meeting the WAO criteria of anaphylaxis^8^ grade 3 through 5. Of 15 patients meeting the criteria of anaphylaxis according to either the Brighton or the WAO criteria, only five patients were identified as anaphylaxis using both tools. Four patients, meeting the Brighton, but not the WAO criteria of anaphylaxis, were suffering from skin symptoms, and subjective upper airway symptoms (*n* = 3), or tachycardia with increased capillary refill time (*n* = 1). Six patients meeting the criteria for anaphylaxis according to WAO, but not to Brighton criteria, were suffering from respiratory symptoms (*n* = 5) or vomiting (*n* = 1) only.

## 4 DISCUSSION

In the entire country of Denmark (5.8 million inhabitants), 36 cases of anaphylaxis according to the Brighton classification (26 PB, 2 M, and 8 AZ), were recorded by the authorities,^13^ during the period (December 27, 2020 to March 31, 2021). When using the Brighton classification, in our region we had nine anaphylactic reactions referred to the Allergy Center (none were immediate Brighton group 1 reactions), out of 199,377 vaccinations administered,^5^ during the same period. We have a close collaboration with the medical departments in the region, and thus rest assured, that we have had all eligible patients referred. Our nine cases of mild to moderate reactions, in 199,377 vaccinations, result in an incidence of 45 per million. Data from the US authorities show incidences between 2.5 and 11.1 per 1 million vaccinations,^2^, ^14^ whereas the first study on diagnostic work up in patients in the United States reports an alarmingly high incidence of 247 cases per million.^15^ These discrepancies remain to be explained.

In a large population based study, vaccines are generally thought to induce anaphylaxis at a rate of 1.31 cases per million doses.^1^ In this study, we only saw one evident case of anaphylaxis (Brighton level 1), and this case was only possibly linked to the PB‐vaccine, since the reaction happened 40 h after vaccination and in combination with intake of NSAID. This indicates that the incidence of anaphylactic reactions to COVID‐19 vaccines is compatible to findings with other vaccines. The low rate of anaphylaxis may be facilitated by the fact that our citizens with the highest probability of developing severe reactions (patients with mastocytosis, patients with known reactions to drug excipients, and patients with prior reactions to other vaccines) were evaluated—and in some cases COVID‐19 vaccinated—in our center.

Another explanation could be that evaluation by the authorities based on written information without assistance of supplemental information from the hospital nor from the patient (preferentially with photos taken by the patient or by relatives) overestimates the incidence as we previously have reported for cases of anaphylaxis in the acute ward.^16^

The majority of the reactions, fulfilling the Brighton level 1 through 3 criteria, was immediate, elicited within 30 min after injection. Same pattern is seen in other studies.^17^ We were able to successfully revaccinate the vast majority of patients, including the patients from Brighton level 2 through 3, indicating, that the majority of patients with reported severe reactions, did not had a true allergic reaction, which is in line with recently published data.^18^ For example, tachycardia, flushing, and subjective transient respiratory symptoms may also be signs of anxiety.

Fifty‐two patients did not meet the case definition of anaphylaxis according to the Brighton criteria. Skin symptoms were predominant, and the majority of patients suffered from late onset reactions ranging from 6 h to several days after vaccination. The mechanism of delayed onset reactions in vaccines are poorly described, and most often, there is no recurrence of symptoms at revaccination.^19^, ^20^, ^21^

Fifty‐five patients had an adverse reaction to first COVID‐19 vaccine. After testing, we were able to revaccinate all but three patients (one patient with positive skin tests to all excipients and two abstaining from revaccination), either with the culprit vaccine, or in the case of reactions to the AZ‐vaccine, which was withdrawn from the Danish market due to side effects, with PB‐ or M‐vaccine instead. No adverse reaction were elicited.

Drug excipients are thought to be the major cause of anaphylaxis to vaccines.^19^, ^20^, ^21^ Only six patients were positive in SPT or BaHR testing to the COVID‐19 vaccines and/or drug excipients:

Three patients were positive in the SPT testing to drug excipients. Two of them were sensitized to PEG at the primary testing. The third patient developed a positive skin test to DMG‐PEG‐2000 after successful revaccination. For safety reasons, all three patients will receive future vaccinations in the Allergy Center.

Three patients were marginally positive to a COVID‐19 vaccine in the BaHR testing; all three were successfully revaccinated. One patient was diagnosed with systemic mastocytosis after reacting to both COVID‐19 vaccinations, pinpointing the importance of special considerations to this patient group.

Based on the results of revaccination, the negative predictive value of SPT and BaHR testing seems high, since no patients with negative test results experienced an allergic reaction to the second vaccine. Positive predictive value, sensitivity, and specificity, however, remains to be determined in larger populations.

In this cohort, both diagnostic scoring systems were able to identify the one patient suffering from true anaphylaxis. The Brighton classification is widely used by the authorities in connection with allergic reactions to drugs including vaccines, whereas the WAO criteria are applied in allergologist settings. While the Brighton criteria focus on the level of diagnostic certainty demanding multiorgan involvement in cases of anaphylaxis,^6^ the WAO criteria focus on the severity of symptoms not demanding multiorgan involvement.^8^ In this study, the concordance between the tools is in line with previous correlations found between different tools for scoring anaphylaxis.^22^ Similar differences have been obtained between the Brighton, Ring and Messmer, and NIAID/FAAN validated scales.^18^ Maybe in the future both scoring systems should be applied for evaluating anaphylaxis to vaccines. The main take home message is however, that the value of diagnostic work up including interview and testing remains superior to written reports.

In conclusion, we showed that anaphylactic reactions in connection with COVID‐19 vaccination are rare. After proper diagnostic work up, it is safe to revaccinate the vast majority of patients with an adverse event to a COVID‐19 vaccine, as most patients with an immediate adverse reaction did not have true allergic reactions. All patients, with late onset hypersensitivity reactions, tolerated revaccination. We, however, diagnosed three patients with drug excipient allergy, stressing the importance of proper evaluation of patients with suspected allergic reactions to vaccines in order to avoid future adverse reactions in these patients. Thus in the majority of cases allergic reactions to the first COVID‐19 vaccine should not prevent the citizen from receiving the second dose, but should prompt allergological testing prior to revaccination. Furthermore, the incidence of allergic reactions to COVID‐19 vaccines seems similar to other virus‐based vaccines.

## CONFLICTS OF INTEREST

The authors declare that they have no conflicts of interest.

## AUTHOR CONTRIBUTIONS

Trine Holm Rasmussen: Data curation; Equal, Formal analysis; Equal, Investigation; Lead, Writing‐original draft; Lead, Writing‐review & editing; Equal, Charlotte G Mortz: Conceptualization; Supporting, Validation; Supporting, Writing‐review & editing; Supporting, Torbjorn Kabel Georgsen: Investigation; Supporting, Writing‐review & editing; Supporting, Helene Marlies Rasmussen: Investigation; Supporting, Writing‐review & editing; Supporting, Henrik Fomsgaard Kjaer: Investigation; Supporting, Writing‐review & editing; Supporting, Carsten Bindslev‐Jensen: Conceptualization; Lead, Funding acquisition; Lead, Investigation; Supporting, Methodology; Lead, Project administration; Lead, Resources; Lead, Supervision; Lead, Validation; Lead, Writing‐review & editing; Equal.
